# Supplementary material for: Bayesian credible sets for phylogenetic tree topologies with applications to coverage analysis and cross-model comparison
Source: Mol Biol Evol. 2026 Jun 11;43(7):msag141. doi: 10.1093/molbev/msag141 (PMC13325671; doi:10.1093/molbev/msag141)
Supplement: msag141_Supplementary_Data [file msag141_supplementary_data.pdf]

Supporting Information to "Bayesian Credible Sets for Phylogenetic Tree Topologies with Applications to Coverage Analysis and Cross-Model Comparison"

Jonathan Klawitter and Alexei J. Drummond

University of Auckland, Aotearoa/New Zealand

Additional Sensitivity and Specificity Analyses

We provide additional sensitivity and specificity results in Figs. 1 to 4.

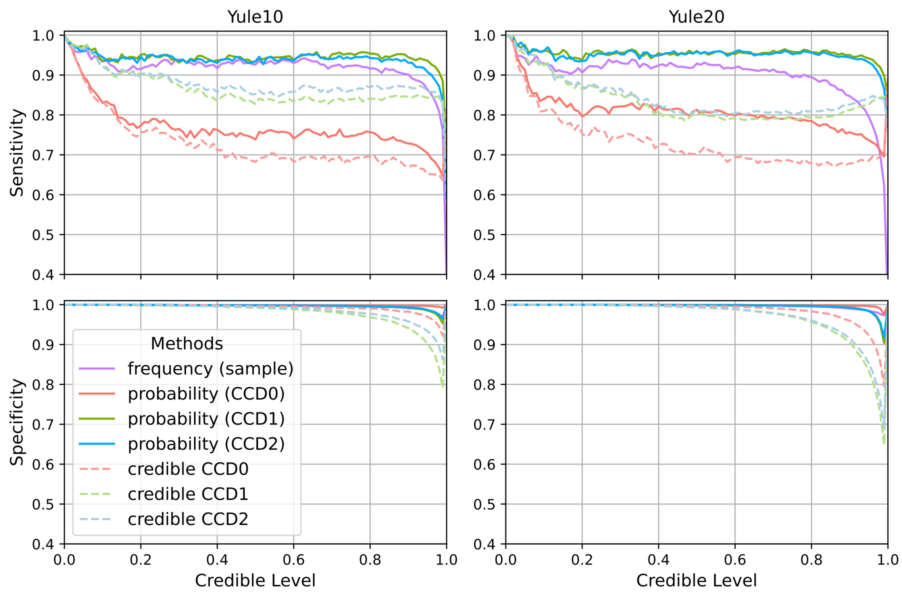

Fig. 1: Mean sensitivity and specificity of the different credible set methods over 250 replicates of `Yule10` and `Yule20` with 3000 trees used in the sample distribution and to build the CCDs. (Note that the y-axes start at 0.4.)

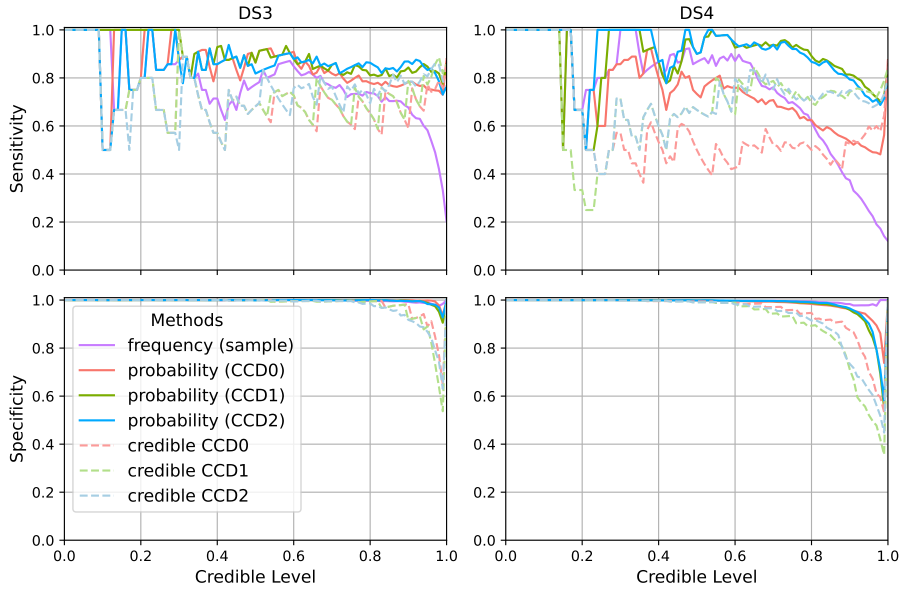

Fig. 2: Sensitivity and specificity of the different credible set methods on `DS3-4` with 3000 trees used in the sample distribution and to build the CCDs.

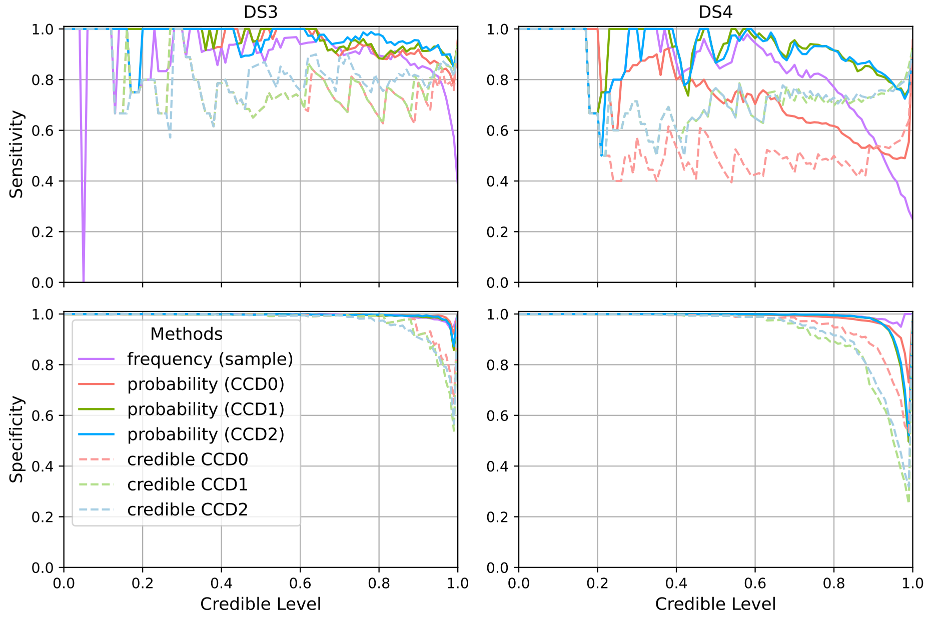

Fig. 3: Sensitivity and specificity of the different credible set methods on `DS3-4` with 10 000 trees used in the sample distribution and to build the CCDs.

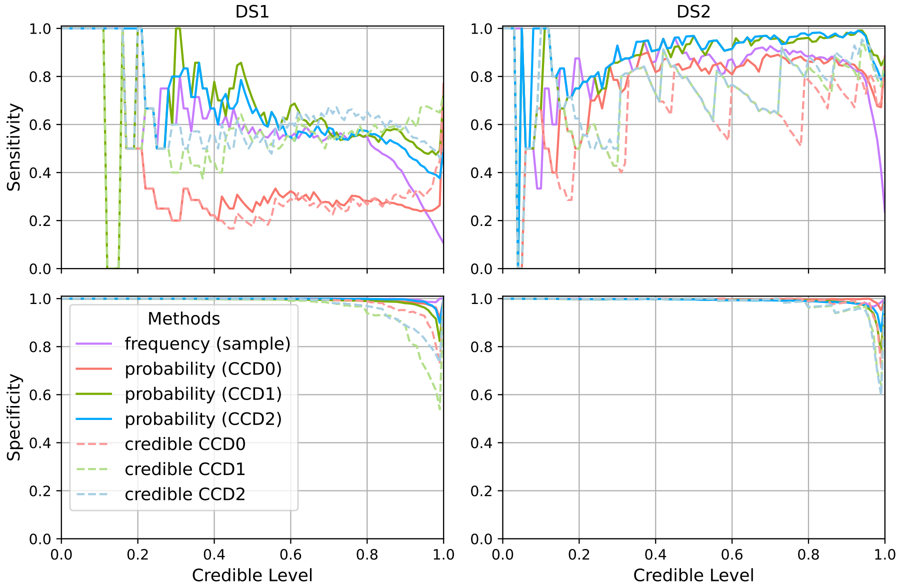

Fig. 4: Sensitivity and specificity of the different credible set methods on `DS1-2` with 10 000 trees used in the sample distribution and to build the CCDs.

Probability-based Method Parameter Choice

We want to demonstrate the effect of the sample size used for probability-based credible sets with a step size of 1%, here with CCD1 and using 100, 1000, 10 000, and 10 000 samples on a `Yule50` simulation. To this end, we sampled random trees with a credible level that is a multiple of 5 from a probability-based CCD1 credible set on 100 000 samples. Then computed credible sets (the probability thresholds) for the different number of samples one hundred times. The difference to mean for each sample size is illustrated in Fig. 5. We conclude that, at least for a `Yule50` dataset, 10 000 samples yield an acceptably small variance.

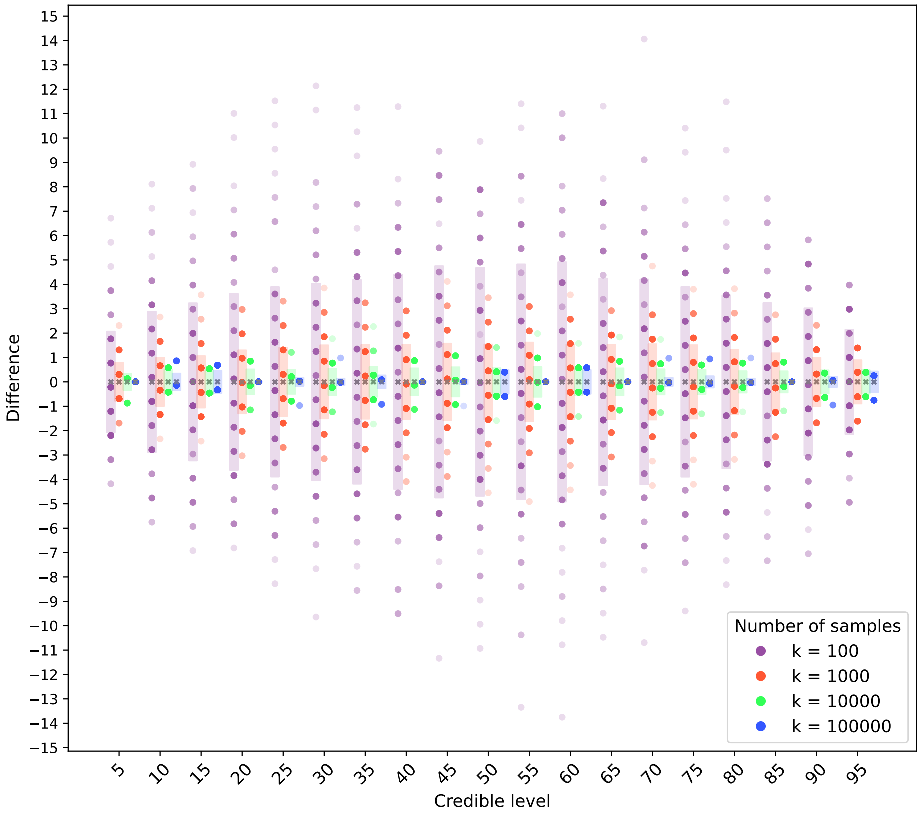

Fig. 5: Difference in the computed credible level of random trees (one per multiple of 5%) by the probability-based credible set method on a CCD1 when using different numbers of samples.

Cross-Model Comparison on Empirical Data

To illustrate a practical application of credible sets, we compared posterior tree topology distributions inferred under two competing evolutionary clock models (Douglas et al., 2025): a gradual clock model and a gradual+abrupt model that additionally allows for instantaneous bursts of evolution at speciation events. We analysed three datasets from that study: a cephalopod morphological dataset (80 taxa), an aminoacyl-tRNA synthetase (aaRS) protein structural dataset (142 taxa), and an Indo-European language dataset (161 taxa). Following Berling et al. (2025), we used CCD0 to obtain point estimates (MAP trees) under each model, and CCD1 to construct split-based credible sets.

In all three datasets, the CCD0 MAP trees of the two clock models differ in topology, with rooted Robinson–Foulds distances of 18/156 (cephalopod), 22/280 (aaRS), and 12/318 (Indo-European). A natural question is whether these topological differences are statistically significant given the posterior uncertainty around each tree. The RF distance alone cannot answer this question, but credible sets can. As shown in Table 1, all MAP trees lie well within the CCD1 credible set of the opposing model: the highest credible level observed is 0.30 (aaRS gradual MAP tree in the gradual+abrupt credible set), meaning that tree is still more probable than 70% of all trees in that posterior. In no case does a MAP tree approach the 95% credible set boundary. The topological differences between the two clock models are therefore not statistically significant in any of the three datasets.

Douglas et al. (2025) noted that the cephalopod clade support differences between the two clock models were "not as extreme" as those observed for the aaRS dataset, and that the aaRS models "produced substantially different clade posterior supports". Our credible set analysis quantifies these qualitative observations at the whole-tree level: while all three datasets show non-significant topological differences, the aaRS MAP trees sit further from the core of the opposing credible set (credible levels up to 0.30) compared to the cephalopod trees (up to 0.08) and the Indo-European trees (up to 0.002). Importantly, even for the aaRS dataset where individual clade support differences were statistically significant, the overall tree topologies are not significantly different.

Table 1: Cross-model credible set analysis. CCD0 MAP trees are used as point estimates; CCD1 split-based credible levels quantify how deep each MAP tree sits in the opposing model's posterior. Lower values indicate the tree is closer to the core of the credible set; a value approaching 1.0 would indicate the tree lies near the boundary. Trees used after 10% burn-in: cephalopod 8551/4009, aaRS 1622/1907, Indo-European 1440/4074.

		CCD1 credible set of	
Dataset	CCD0 MAP tree from	gradual	gradual+abrupt
Cephalopod (RF = 18/156)	gradual	0.00	0.08
	gradual+abrupt	0.00	0.00
aaRS (RF = 22/280)	gradual	0.00	0.30
	gradual+abrupt	0.01	0.00
Indo-European (RF = 12/318)	gradual	0.00	0.00
	gradual+abrupt	0.00	0.00

Bibliography

Lars Berling, Jonathan Klawitter, Remco R. Bouckaert, Dong Xie, Alex Gavryushkin, and Alexei J. Drummond. Accurate Bayesian phylogenetic point estimation using a tree distribution parameterized by clade probabilities. *PLOS Computational Biology*, 21(2):1–21, 2025. https://doi.org/10.1371/journal.pcbi.1012789.

Jordan Douglas, Remco Bouckaert, Simon C Harris, Charles W Carter Jr., and Peter R Wills. Evolution is coupled with branching across many granularities of life. *Proceedings of the Royal Society B*, 292:20250182, 2025. https://doi.org/10.1098/rspb.2025.0182.
